# The influence of the combined treatment with Vadimezan (ASA404) and taxol on the growth of U251 glioblastoma xenografts

**DOI:** 10.1186/1471-2407-12-242

**Published:** 2012-06-13

**Authors:** Dušan Milanović, Friederike Braun, Wolfgang Weber, Anca Ligia Grosu, Martin Behe, Gabriele Niedermann

**Affiliations:** 1 Department of Radiation Oncology, University Hospital Freiburg, Freiburg 79106, Germany; 2 Department of Nuclear Medicine, University Hospital Freiburg, Freiburg 79106, Germany; 3 Paul Scherer Institut, Center for Radiopharmaceutical Sciences, Wolfgang-Pauli Strasse 10, Zurich CH-8093, Switzerland; 4 University Hospital Freiburg, Department of Radiation Oncology, Robert Koch Strasse 3, Freiburg D-79106, Germany

## Abstract

**Background:**

One of the most important biological characteristics of Glioblastoma multiforme (GBM) is high vascular density. Vadimezan (ASA404, DMXAA) belongs to the class of small molecule vascular disrupting agents (VDA) that cause disruption of established tumor vessels and subsequent tumor hemorrhagic necrosis. Its selective antivascular effect is mediated by intratumoral induction of several cytokines including tumor necrosis factor-α (TNF-α), granulocyte-colony-stimulating factor (G-CSF), interleukin 6 (IL-6) and macrophage inflammatory protein 1α (MIP-1α). Preclinical studies have demonstrated that ASA404 acts synergistically with taxanes. In this study, we investigated if treatment of mice bearing U251 human glioblastoma xenografts with ASA404 and taxol may be synergistic. Therapy response was evaluated by measuring changes in tumor size and metabolic activity using ^18^F-FDG PET (Fluorodeoxyglucose - positron emision tomography) imaging.

**Methods:**

U251 cells were inoculated s.c. in the right hind limb of NMRI-Foxn1^nu^ athymic female nude mice. Animals were randomly assigned into 4 groups (7–9 animals/group) for treatment: control, taxol, ASA404, and ASA404 plus taxol. The animals received either a single dose of taxol (10 mg/kg), ASA404 (27.5 mg/kg), or taxol (10 mg/kg) plus ASA404 (27.5 mg/kg) administered i.p.; ASA404 was administred 24 h after the treatment with taxol. 4 and 24 h after treatment with ASA404 (28 and 48 h hours after treatment with taxol) ^18^ F-FDG PET scans were performed.

**Results:**

The treatment with taxol did not affect the tumor growth in comparison to untreated controls. The treatment of animals with single dose ASA404 alone or in combination with taxol caused a significant delay in tumor growth. The combined treatment did not decrease the growth of the xenografts significantly more than ASA404 alone, but early changes in tumor ^18^ F-FDG uptake preceded subsequent growth inhibition. The tumor weights, which were determined at the end of treatment, were lower in case of combined treatment.

**Conclusions:**

The treatment with ASA404 alone or in combination with taxol showed antitumoral effects in our glioblastoma model probably through destruction of blood vessels. The implications for the anticancer effect of this compound warrant further preclinical studies. ^18^F-FDG PET appears to be a promising tool to monitor treatment with ASA404 early in the course of therapy.

## Background

Glioblastoma multiforme (GBM) is the most common brain tumour of astrocytic origin [1]. Despite the use of multimodal therapy (surgery and radiochemotherapy with Temozolomide) the prognosis of patients with this highly aggressive neoplastic disease remains poor with a median survival time of 14.6 months [2]. One of the most important biological characteristics of GBM is high vascular density. Thus targeting the vasculature in this tumour could be an attractive therapeutic strategy [3]. Bevacizumab is a monoclonal antibody against VEGF (Vascular Endothelial Growth Factor), which is one of key proteins stimulating the growth of new blood vessels (angiogenesis) in glioblastomas [4]. This drug was granted accelerated approval by the US Food and Drug Administration (FDA) as a single agent in recurrent GBM [5].

5,6-Dimethylxanthenone-4-Acetic Acid (ASA404, DMXAA) belongs to the class of small molecule vascular disrupting agents (VDA) that, differently to antiangiogenic agents, cause disruption of established tumor vessels by induction of apoptosis in endothelial tumour cells what is followed by rapid vascular collapse and subsequent tumor hemorrhagic necrosis [6]. The mechanism of the selective antivascular effect of ASA404 in the tumor vasculature is not well understood but it has been proposed that these effects are mediated by intratumoral induction of several cytokines including tumor necrosis factor-α (TNF-α), granulocyte-colony-stimulating factor (G-CSF), interleukin 6 (IL-6), macrophage inflammatory protein 1α (MIP-1α) [7,8], increased plasma serotonin concentration [9] and induction of intratumoral nitric oxide synthase [10]. In addition to antivascular effects, ASA404 has an antiangiogenic effect mediated by induction of the antiangiogenic chemokine interferon-inducible protein 10 (IP-10) which inhibits basic fibroblast growth factor-induced neovascularization in several models *in vitro* and *in vivo*[11]. Preclinical studies have demonstrated that ASA404 acts synergistically with severeral conventional chemotherapeutic agents. The most pronounced synergistic effect was observed when ASA404 was combined with taxane [12]. Taxol is a mitotic inhibitor which is used in the therapy of many neoplastic diseases. It binds and stabilizes microtubules by inhibiting tubulin depolarization and causing cell cycle arrest in the G2/M Phase [13]. In addition to its cytostatic effect on cancer cells, taxol (at lower concentrations) inhibits endothelial cell function related to angiogenesis [14]. It has been shown that taxol delays growth of human GBM xenografts [15] and we hypothesized that combined treatment with ASA404 may be synergistic in mice bearing U251 human glioblastoma xenografts. An additional aim of thisstudy was to examine if non-invasive imaging using ^18^ F-FDG – PET (Fluorodeoxyglucose–positron emision tomography) may allow early assessment of tumor response to therapy.

## Methods

### Animals

All animal experiments were performed in accordance with the German Animal License Regulations and were approved by the animal care committee of the Regierungspräsidium Freiburg (registration number: G-08-59). Athymic female nude mice NMRI-Foxn1^nu^ (aged 6 weeks) were obtained from Taconic Europe (Bomholtgard, Denmark) and tumors were induced by inoculation of 1.5 × 10^6^ human glioblastoma (U251) cells s.c. into the right hind limb. Palpable U251 xenografts developed within 8 to 10 days.

### Drug treatment

ASA404 was kindly provided by Novartis Pharma AG Basel, Switzerland. ASA404 powder was dissolved in phosphate buffered saline directly prior to administration. Stock solution of taxol (Sigma-Aldrich, Taufkirchen, Germany) was prepared using dimethylsulfoxide (DMSO) as the vehicle and appropriately diluted in saline before treatments. Animals with 10 days-old xenografts were randomly assigned in 4 groups (7–9 animals/group) for treatment: control, taxol, ASA404 and ASA404 plus taxol. The animals received either a single dose of taxol (10 mg/kg), ASA404 (27.5 mg/kg), or taxol (10 mg/kg) plus ASA404 (27.5 mg/kg) administered i.p.; ASA404 was administred 24 h after the treatment with taxol. Animals in the control group were treated with DMSO i.p. Animal weight was measured daily. In order to determine tumor volume by external caliper, the greatest longitudinal diameter (length) and the greatest transverse diameter (width) were determined. Tumor volume based on caliper measurements was calculated by the modified ellipsoidal formula:

(1) $$Tumor\;volume=\frac{1}{2}\;\left(length\times width^{2}\right)$$

15 days after initiation of treatment animals were sacrificed following the IACUC guidelines.

Xenografts were surgically excised and their weights were measured.

### PET scanning

PET imaging was performed with a microPET focus 120 small animal PET scanner (Concorde, Knoxville, TN). After 4–8 h of fasting, 7.4 MBq ^18^ F-FDG was injected via a lateral tail vein. For ^18^ F-FDG injection and imaging the mice were anesthesised with 1.5–2% Isoflurane. PET imaging was started 60 min after injection. Image acquisition time was 10 min. Images were corrected for dead-time and randoms and reconstructed by the routine OSEM2D (2-Dimensional Reconstruction) algorithm provided by the manufacturer. Image counts per pixel per second were calibrated to activity concentrations (Bq/ml) by measuring a 3.5-cm cylinder phantom filled with a known concentration of ^18^ F-FDG [16,17]. The resolution of the reconstructed images ranged between 1.5-2 mm depending on the distance from the center of the field of view [18]. ^18^ F-FDG uptake by the tumor xenografts, brain and liver was quantified by maximum standardized uptake values (SUVs) normalized to the weight of the animals. Quantitative analysis was performed with the Amide software [19]. In order to identify the maximum tumor ^18^ F-FDG uptake large ellipsoid regions of interests were drawn around the whole tumor area and organ, respectively. The voxel with the maximum ^18^ F-FDG uptake was then identified automatically by the Amide software.

### Statistical analysis

Mann–Whitney *U* test was used to compare quantification data. Statistical analysis was conducted with Statistical Package for Social Sciences software (SPSS Inc.). We used a 2-sided significance level of 0.05 for all statistical analyses.

## Results

### Tolerability of treatment

**Figure 1:**
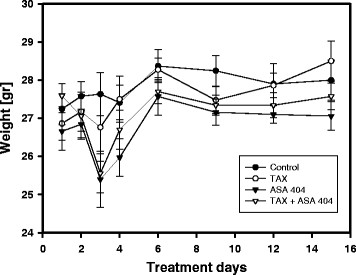
**The effect of taxol and ASA404 as sole agents or in combination on body weight of mice.** One day after treatment with ASA404 alone or in the combination with taxol temporary weight loss of animals was observed. Three days after treatment with ASA 404, animal body weight had normalised compared to the control group or the group which was treated only with taxol. By the end of observation period, no significant difference in body weight between control group and treated animals was observed.

The treatment with taxol as a sole compound was well tolerated. No side effects were observed. In animals, which were treated with ASA404 alone or in combianation with taxol, slight diarrhea and significant weight loss were observed after 1 day of treatment with ASA404 with full recovery by 3 days [Figure 1]. Weights of animals remained relatively constant for the rest of the experimental period.

### Activity of ASA404 and taxol on the growth and weight of U251 human glioblastoma xenografts

**Figure 2:**
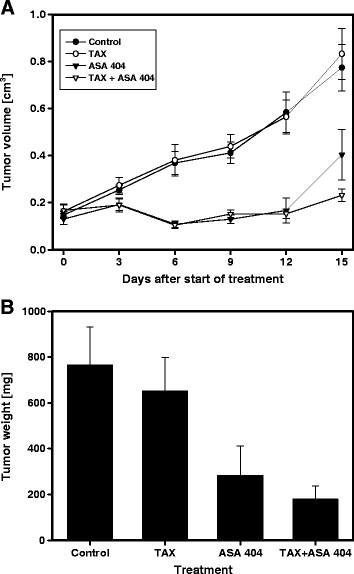
**The effect of taxol and ASA404 as sole agents or in combination on xenograft volume at different time points** [**A**] **and weight** [**B**] **at the end of treatment.** The treatment of animals with taxol did not affect xenograft growth in comparison to untreated controls. Treatment with ASA404 alone or in combination with taxol caused significant growth delay of tumors in comparison to control or animals which were treated with taxol as a sole compound. There was not significant difference in tumor growth delay between animals which were treated with ASA404 alone or in combination with taxol. On the end of the treatment, the tumor weight of animals treated with the ASA404 and taxol combination were significantly lower than from animals which were treated only with ASA 404.

**Figure 3:**
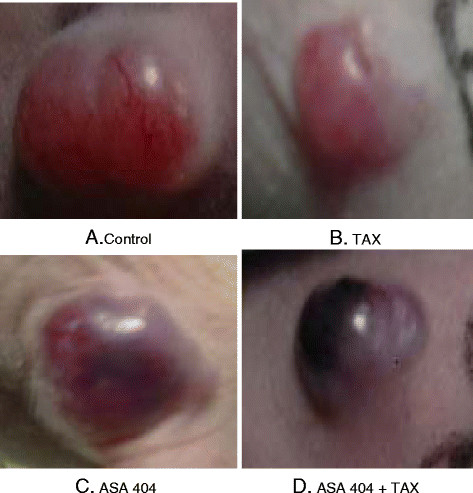
**Color change of xenografts 32 h after the treatment with taxol** [**B**]**, 8 h after the treatment with ASA404** [**C**] **or 32 h after the treatment with taxol plus a ASA404 during the last 8 h** [**D**]**. Xenograft** [**A**] **represents an untreated control.** Treatment with ASA404 as a sole agent or in combination with taxol most probably caused change of permeability and selective disruption of blood vessels within tumors followed by hemorrhagic necrosis.

The treatment with taxol did not affect the tumor growth in comparison to untreated controls. The treatment of animals with single dose ASA404 alone or in combination with taxol caused a significant decrease in tumor volume [Figure 2A]. At the completion of the study, weights of the excised tumors were: control = 764 ± 168 mg, taxol = 651 ± 148 mg, ASA404 = 283 ± 127 mg, ASA404 +taxol =180 ± 56 mg. ASA404 as a sole agent caused a significant decrease in tumor weight. In case of combined treatment, statistically significant lower tumor weight was observed compared to treatment with ASA404 as a single agent (p = 0.0198) [Figure 2B]. Already 8 h after treatment, xenografts in animals treated with ASA404 alone [Figure 3C] or in combination with taxol [Figure 3D] remarkably changed color. This change was not observed in untreated animals [Figure 3A] or in animals which were treated only with taxol [Figure 3B].

### PET

**Figure 4:**
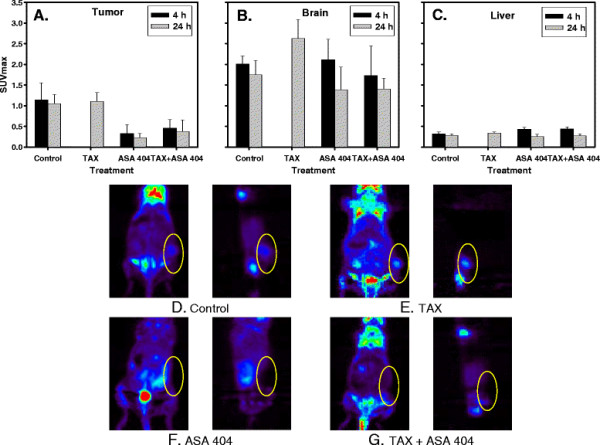
**SUV for tumor** [**A**]**, brain** [**B**] **and liver** [**C**] **4 and 24 h after treatment with ASA 404.** Small animal FDG-PET imaging (frontal and saggital sections) 4 h after treatment with taxol [**E**], ASA404 [**F**] or TAX + ASA404 [**G**]. [**D**] represents an untreated control. The treatment of animals with ASA404 caused a marked decrease of the ^18^ F-FDG uptake which existed also 24 h after treatment with same compound. In case of combined therapy the same effect was observed. No significant change of ^18^ F-FDG uptake was observed in the brain [Figure 4B] and the liver [Figure 4C] in ASA404 treated animals in comparison to untreated control. The treatment with taxol as a sole compound did not influence significant change of SUVs.

The treatment with taxol did not affect the ^18^ F-FDG uptake in comparison to untreated controls. However, the treatment of animals with ASA404 caused a rapid and marked decrease in tumor ^18^ F*-*FDG uptake already 4 h after treatment with ASA404 (p = 0.0495 - three mice in each group). There was a similar decrease in ^18^ F-FDG uptake at 24 h after treatment with ASA404 (p = 0.0455) The combined therapy with ASA404 and taxol showed significant reduction of the ^18^ F-FDG uptake, too, comparable to the one observed with ASA404 alone. Between 1–12 h after this investigation, several mice treated with ASA404 alone or in combination with taxol died. However, no significant change of ^18^ F-FDG uptake was observed in the brain [Figure 4B] and liver [Figure 4C] in ASA404 treated animals in comparison to untreated control. No mice died in groups which were not used for PET studies and which were treated with the same drugs respectively, but a transient significant weight loss was observed in all ASA404 treated animals.

## Discussion

Despite intensive research and development of new targeted therapies and radiotherapeutic techniques, prognosis for patients with GBM remains poor indicating the need for new therapeutic approaches. Because of high vascular density of these tumors development of therapies selectively targeting the tumor vasculature may be meaningful. The aim of this study was to identify if the combined treatment with ASA404 and taxol shows synergistic effects in mice bearing U251 human glioblastoma xenografts and if non-invasive ^18^ F-FDG PET imaging could be used to monitor tumor response early in the course of therapy. Tumors of animals which were treated with ASA404 as a sole agent or in combination with taxol showed significant growth delay in comparison with control or animals which were treated only with taxol. Combined treatment with taxol and ASA404 had a similar effect on tumor growth as ASA404 alone. At the completion of the study, there was only slight but statically significant difference in tumor weight for animals treated with the drug combination as compared to animals receiving ASA404 alone. This difference in statistical significance between the two parameters which were analysed (tumor volume and tumor weight) can be explained by the use of a formula in which one important parameter of the tumor growth – tumor depth, was not considered. The tumor weights were determined only at the end of treatment and in this may represent a more precise parameter to determine an effect of the therapy. On the other hand, the differences among treatment groups were very small. Thus, based on these data, it is difficult to conclude if combined treatment was synergistic. GBM represents one of the most vascularized tumors [20]. Antiangiogenic agents, such as bevacizumab, which is approved for treatment of recurrent GBM patients who have failed previous temozolomide and radiation therapy, inhibit new blood vessel formation from preexisting vasculature [5]. Furthemore, antiangiogenic agents can normalize the tumor vasculature and decrease interstitial fluid pressure, providing an improved drug delivery [21].

In contrary to antiangiogenic agents, VDAs such a ASA404, have a selective affinity to existing tumor blood vessels inducing their collapse which impairs blood flow, oxygen supply and consequently causes necrosis in tumor tissue [22].

The synergistic mechanism between ASA404 and taxol seems not to involve potentiation of the vascular disrupting activity of ASA404 or a pharmacokinetic interaction between these two drugs. The synergism might be explained by complementary action against the different subregions of the tumor; ASA404 is more active in the poorly vascularized regions while taxol is active in the well vascularized regions [12]. In addition to its direct cytotoxic effects, taxol can induce the expression of pro-inflammatory cytokines, such as TNF-α and IL-6 [23] which are important mediators of ASA404 activity [7,8]. Thus, taxol and ASA404 can induce of same cytokines which are responsible for tumor vascular disruption.

It has been reported that chemotherapy drugs should be administered before, or shortly after ASA404 in order to avoid compromised delivery. When taxol was administred 4 h after ASA404, considerable loss of antitumor activity has been observed apparently caused by decreased blood flow which may inhibit taxol distribution in tumor tissue [12].

As opposed to conventional antineoplastic agents, effects of VDAs such as ASA404 do not result in dramatic changes in tumor volume [22,24]. This indicates that new approaches are necessary to monitor tumor response to VDAs. We found that ^18^ F-FDG uptake decreased rapidly after administration of ASA404. This result is consistent with findings that highest increase of intratumoral TNF-α activity is also observed 4 h after tratment with ASA404 [25]. We suppose that the marked reduction in tumor ^18^ F-FDG uptake is at least partly a reflection of tumor cell death. Moreover, it seems likely that a significant fraction of tumor cells will become necrotic, if perfusion is decreased to such an extent that ^18^ F-FDG uptake significantly decreases. Preclinical studies in different tumor models have demonstrated that ASA404 directly disrupts the tumor vasculature by selectively inducing apoptosis in tumorvascular endothelial cells [26,27]. On the other hand, the reason why several of the mice who underwent PET imaging died within several hours after the PET scan is not entirely clear. Because of ASA404 antivascular properties, we presumed that bleeding might cause this effect. Suprisingly, mouse dissection did not show any bleeding source. There is only one report on the use of small animal PET as a biomarker for response to ASA404 [28]. In this case, ^18^ F-fluromisonidazole (FMISO) PET imaging was conducted and no side effects have been reported. Before application of the ^18^ F-FMISO tracer, the animals do not have to fasten.

It has been shown that the administration of TNF-α, which is an important mediator of ASA404 antitumoral action, decreases serum glucose levels in mice [29]. For improving tumor visualization, mice have to fasten a ^18^ F-FDG injection uptake period [30]. We speculate that the combination of ASA404 mediated TNF-α secretion with starving and anesthesia for PET investigation may have caused death of mice.

It has been reported that treatment of nude mice bearing U87 and GL261 orthotopically grown human glioblastoma cells with ASA404 caused statistically significant increase in median survival compared to untreated controls [31]. Measurement with contrast-enhanced magnetic resonance imaging (MRI) and diffusion-weighted MRI which were used to determine tumor blood flow 24 h after treatment with ASA404 clearly demonstrated extravasation and accumulation of the contrast agent in the tumor indicating treatment-induced vascular disruption. Similar effects were observed in fibrosarcoma (MCA205) ectopic and orthotopic tumor models. Remarkably, 3 h after treatment with ASA404 ectopic tumors showed 6-fold greater induction of TNF-α compared to orthotopic tumors [32]. In our glioblastoma U251 model, the rapid mode of action of ASA404 became also apparent by the changes in tumor color 8 h post injection which indicates hemorrhagic necrosis.

It is believed that VDAs are more effective against vessels inside of the tumor. In the periphery of the tumor a characteristic rim of cells will remain viable after treatment [33,34].

In our experiments, tumors of animals which were treated with ASA404 as a sole agent or in combination with taxol, showed significant growth delay in comparison with control or animals which were treated only with taxol. In both group of ASA404-treated animals, tumor start to regrowth between 11 und 13 days after treatment.

Despite the significant smaller tumors, 20 days after treatment, histopathologic examination revealed that all treated tumors maintained the characteristic growth of glioblastoma without any difference between the different treatment groups (data not shown). No significant increase in the level of cell death occured in tumors of animals treated with ASA404 indicating that surviving tumor cells were able to proliferate causing tumor regrowth. In the Colon 38 adenocarcinoma tumor model [35], the antitumor effect of ASA404 was schedule-dependent. 100% of the tumors regressed, when the mice were treated with loading (25 mg/kg) and two supplementary (5 mg/kg) doses after 4 and 8 h while in mice treated with a single dose of ASA404 (25 mg/kg) 40% of tumors regressed. We supose that with a prolonged therapy better antitumoral responses might be achieved. The first clinical data obtained from I/II phase clinical trials evaluating ASA404 in combination with taxol and carboplatin in patients with untreated advanced non-small lung cancer were promising [36] but in one large, randomized phase III placebo controled trial, these results were not confirmed [37].

## Conclusions

We conclude that treatment with ASA404 showed significant antitumoral effects in our glioblastoma model. This warrants further preclinical studies of ASA404 in other glioblastoma models and in combination with conventional antineoplastic drugs such are temozolamide and carmustine standardly used in the treatment of glioblastoma. Further understanding the mechanisms of VDAs for treatment optimization is necessary. If the toxicity associated with the ^18^F-FDG PET examination would not occur in tumor patients, ^18^F-FDG PET could be a promising tool to monitor the drug effects in the clinic early in the course of therapy.

## Competing interests

Authors declare that there are no financial interests in relation to the work described.

## Authors’ contributions

DM was the principal investigator of the study and largely wrote the manuscript. FB and MB designed and conducted small animal PET experiments. WAW analysed PET experiments and participated in writing the manuscript. ALG provided clinical information and participated in the design of the study. GN supervised the research, analysed and interpreted data, and participated in writing the manuscript. All authors read and approved the final manuscript.

## Pre-publication history

The pre-publication history for this paper can be accessed here:

http://www.biomedcentral.com/1471-2407/12/242/prepub
